# Physical Activity and Plasma Glucose Control among Diabetic Patients Attending Outpatients Clinics in Hanoi, Vietnam

**DOI:** 10.3390/ijerph18031182

**Published:** 2021-01-29

**Authors:** Tam Ngoc Nguyen, Tam Thi Nguyen, Maria Hagströmer, Thang Pham, Ingeborg van der Ploeg, Carl Johan Sundberg, Huyen Thi Thanh Vu

**Affiliations:** 1 Department of Geriatrics, Hanoi Medical University, Hanoi 100000, Vietnam; ngoctam@hmu.edu.vn; 2 Scientific Research Department, National Geriatric Hospital, Hanoi 100000, Vietnam; phamthang@hmu.edu.vn; 3 Dinh Tien Hoang Institute of Medicine, Hanoi 100000, Vietnam; 4 Outpatient Department, Dong Anh General Hospital, Hanoi 100000, Vietnam; nguyentam.bvda@gmail.com; 5 Department of Neurobiology, Care Sciences and Society, Karolinska Institutet, 141 83 Huddinge, Sweden; maria.hagstromer@ki.se; 6 Academic Primary Health Care Centre, Region Stockholm, 113 65 Stockholm, Sweden; 7 Department of Physiology and Pharmacology, Karolinska Institutet, 171 65 Stockholm, Sweden; ingeborg.van.der.ploeg@ki.se (I.v.d.P.); carl.j.sundberg@ki.se (C.J.S.); 8 Department of Learning, Informatics, Management and Ethics, Karolinska Institutet, 171 65 Stockholm, Sweden

**Keywords:** glucose control, GPAQ, physical activity domain, self-report

## Abstract

Reaching the recommendation on physical activity (PA) for health is highly important to effectively manage blood glucose in patients with type 2 diabetes (T2D). The aims of this study were to assess the level and pattern of PA among T2D outpatients and to relate PA levels to glucose control. A cross-sectional study was conducted in outpatient clinics in Hanoi, Vietnam. PA levels were reported using the Global Physical Activity Questionnaire (GPAQ) version 2.0. Participants meeting the WHO recommendations on PA for health or not were respectively categorized as “sufficiently active” and “insufficiently active”. FPG < 7.2 mmol/L was defined as controlled plasma glucose. In total, 407 participants with T2D (55% women, mean (SD) age 61.6 (9.7) years) were included. The fraction of T2D outpatients reporting as insufficiently active was 21%. The lowest amount of energy expenditure was from transport activities (travel from and to places). On multivariate logistic regression, being sufficiently active was associated with a two-fold increased likelihood of having better glycemic control. The findings warrant action plans to increase physical activity in general and in specific active transport for T2D patients in Vietnam.

## 1. Introduction

Diabetes mellitus is the seventh leading cause of death worldwide [1]. The number of people with type 2 diabetes (T2D) is expected to rise from 451 million in the year 2017 to approximately 700 million by 2045 with the highest rise and total number of patients in low- and middle-income countries [2].

Similarly to what is happening in most lower-middle income countries, the prevalence of T2D in Vietnam has shown a significant rise [3] and was estimated to be 6.0% in community screening surveys carried out during 2011–2013 in northern Vietnam [4]. Previous studies indicated that low physical activity (PA) is increasing rapidly worldwide. In a review from 122 countries, about 31% of adults are insufficiently active [5]. The increase in the incidence of T2D is thought to be strongly related to rapid changes in lifestyle including the adoption of unhealthy food consumption patterns, smoking and a reduction of PA levels [6,7]. People with total PA at least of 600 MET min per week had a 2% lower risk of diabetes in comparison with those reporting no PA [8]. Regular PA and healthy dietary habits are very important to decelerate the incidence and manifestations of T2D [9,10].

PA includes all movements that increase energy use. PA has shown to improve glucose control to the same extent as pharmacological treatment in participants with T2D [11]. Insulin resistance and oxidative enzymes can be increased by aerobic training. There is now ample evidence that reaching the recommended levels of aerobic PA reduces the risk of premature death and chronic diseases such as T2D, lipid disorder, hypertension, cardio-respiratory diseases, osteoporosis, depression as well as breast and colon cancer [12]. It was found in a previous study that aerobic exercise at moderate or high volume are associated with lower mortality risks in diabetic patients [13]. In addition, body weight, waist circumference and blood pressure as well as dyslipidemia (also related to the metabolic syndrome) could be positively influenced by regular PA [11].

PA and exercise should be recommended and prescribed to all individuals with type 2 diabetes [14]. The dose and types of PA and activities for diabetic participants which can reduce HbA1c and glucose are almost the same as the recommendations for the general population [11,14]. The recommendations affirm the importance of regular aerobic and muscle-strengthening activities. The global recommendations on PA for health published by the World Health Organization (WHO, 2010) specified that adults should perform at least 150 min per week of moderate-intensity aerobic PA or 75 min per week of vigorous-intensity aerobic PA or an equivalent combination of moderate- and vigorous-intensity PA [15]. These were the recommendations used at the time of data collection and analysis in the present study. The new WHO recommendations released in November 2020 state that all adults should undertake 150–300 min of moderate-intensity, or 75–150 min of vigorous-intensity PA, or some equivalent combination of moderate-intensity and vigorous-intensity aerobic PA, per week [16].

There is a strong need for fundamental knowledge about PA in T2D participants in the lower middle-income country Vietnam to inform both policy and action plans for the health care system and the general population. The outcome of the study could be valuable as the basis for further studies and especially for facilitation of implementation of recommendations of PA for patients with T2D in Vietnam. The aims of the present study were to assess the level and pattern of PA among T2D study participants in out-patient clinics in Hanoi, Vietnam and to relate PA levels to glycemic management.

## 2. Materials and Methods

### 2.1. Study Population

The study was designed as a cross-sectional investigation, conducted in outpatient clinics of two hospitals, in Hanoi, Vietnam. The National Geriatric Hospital (NGH), which is a leading hospital in Northern Vietnam, started the national program on management of T2D in 2005. Patients referred to NGH (located in a central urban area) and to Dong Anh General Hospital (located in a rather populated rural area) for diagnosis of T2D are upon diagnosis requested to visit every month for checkup and medicine dispensing, which is covered by medical insurance. Patients diagnosed with T2D by using WHO 2006 criteria were consecutively recruited into the study from May 2014 to August 2015 [17]. Exclusion criteria were (1) severe illness, (2) blindness or deafness, (3) severe dementia or delirium, (4) inability to answer the questionnaire, (5) inability to provide consent or refusal to participate in the study.

The protocol of the study was approved by the Ethics Committee of National Geriatrics Hospital, Vietnam (ethic code number of the project: 144/IRB-NGH). T2D participants attending the outpatient clinics were informed in writing about the objective of the study. The participants were interviewed by well-trained surveyors. Data were collected from medical records and patient examination using a predefined data collection form (added as Supplemental Material).

### 2.2. Sample Size Calculation

The sample size was determined using a single population proportion formula: *n* = Z^2^_1−α/2_ * [p*(1 − p)/d^2^], with *n* = the required sample size, Z_1−α/2_ = 1.96 (with α = 0.05 and 95% confidence interval), p = prevalence of diabetic patients having recommended level of PA and d = precision (assumed as 0.05). A study on diabetic patients in Vietnam showed that 59.2% of patients with T2D met the minimum recommended level of PA [18]. This means that the sample size for our study needed to be at least 371 participants.

### 2.3. Physical Activity Level and Pattern Assessment

PA levels and sedentary time were reported and registered in a face-to-face interview using the Global Physical Activity Questionnaire (GPAQ) version 2.0. GPAQ, which was intended as an improvement of International Physical Activity Questionnaire (IPAQ) and relevant to developing countries [19] has been previously validated and used to assess PA in Vietnamese population [20,21]. The questionnaire covers the frequency (days per week) and duration (hours or minutes per day) spent doing moderate- and/or vigorous-intensity PA in three domains: activity at work, travel to and from places and in leisure time; and sedentary time in a typical day. All data processing followed the GPAQ analysis protocol [22].

### 2.4. Categorization of Physical Activity Levels

To calculate total activity energy expenditure, in terms of Metabolic Equivalent of Task (MET), i.e., the ratio of a person’s working metabolic rate relative to resting metabolic rate was used [23]. The energy cost of sitting quietly is defined as 1 MET and equivalent to a caloric consumption of ~1 kcal/kg/hour [22]. When using GPAQ, 4 METs were assigned to the time spent in moderate-intensity activities and 8 METs in vigorous-intensity activities [22]. The total PA score was computed as the sum of all MET minutes per week from physical activities performed in work, transport and leisure time.

The level of PA was categorized by summarizing time spent on moderate-to-vigorous physical activities (MVPA) from all domains to total activity energy expenditure [8,22]:

(1) Moderate level, high level and very high level were defined as achieving 600–2999 MET min per week, 3000 to 7999 MET min per week and minimum 8000 MET min per week, respectively. This corresponds to meeting the WHO recommendation of 150 min of moderate intensity (4 MET) physical activity per week and was categorized as “sufficiently active”.
(2) Low level was defined as achieving less than 600 MET min per week or having sedentary lifestyle. This level corresponds to not meeting the WHO recommendation and was defined as “insufficiently active”.

### 2.5. Sedentary Time

The sedentary behavior question (Items 16) was used to estimate the total time on a typical day spent sitting or reclining (hours and minutes per day). The mean (median) was used to presented sedentary time of study participants.

### 2.6. Sociodemographic and Clinical Factors

Sociodemographic information, such as age and gender (male/female) were obtained. Age was classified as 4 groups: 30–54, 55–64, 65–74, 75–89 years old.

Other clinical outcomes such as body weight and height, waist and hip circumference were measured twice in each individual and the mean was used for the purpose of analysis.

Weight (kg) was measured using an electronic scale (Electronic Body Scale TCS-200-RT), in standing position barefoot and with minimal clothing. Weight was recorded to the nearest 0.1 kg. Height (m) was measured against a convenient flat wall. Participants were standing barefoot and height was recorded to the nearest 0.1 cm. Body Mass Index (BMI) (kg/m^2^) was calculated as weight (kg) per square of height (m^2^).

Waist circumference was measured mid-way between the lower rib margin and the iliac crest and hip circumference was measured at the broadest circumference around the buttock. Waist-Hip Ratio (WHR) was calculated as waist circumference (cm) divided by hip circumference (cm).

Systolic and diastolic blood pressure was measured twice times in a sitting position after the participant rested for at least 5 min. The higher values were used to be analyzed in the study.

Fasting plasma glucose (FPG) was tested after at least 8 h after the last meal. Participants with FPG lower than 7.2 mmol/L were defined as having controlled plasma glucose [24].

### 2.7. Statistical Analysis

The data was analyzed using SPSS 20.0 software. Quantitative variables were expressed as a mean, standard deviation or median. Categorical variables were expressed as absolute and relative frequencies (percentages). To test the difference of sociodemographic and clinical variables in relation to levels of PA, the unequal variances t-test and the Chi-squared test were used. Univariate and multivariate logistic regression were conducted to investigate the relationship between PA levels and management of plasma glucose (Adjusted by selected factors age, gender, family history of T2D, WHR, sedentary time, duration of diabetic diagnosis). The significance level was set at 0.05.

## 3. Results

Four hundred and forty-eight participants were asked to participate in the study. Twenty-nine (6.5%) participants declined to participate. Twelve participants (2.7%) were excluded due to missing information on any domain of PA. Thus, the final study population comprised 407 participants with T2D (55% women, mean (SD) age 61.6 (9.7) years.

The study population’s characteristics are presented in Table 1. In the sufficiently active group, mean age was 62.0 ± 9.5 years and male accounted for 45.2%. In the insufficiently active group, mean age was 59.4 ± 10.4 years and 57.0% were male. Waist hip ratio (WHR) was significantly lower in the sufficiently active group than in the insufficiently active group (0.92 ± 0.05 compared to 0.94 ± 0.05, *p* < 0.05). The mean of BMI was in normal range and there was no considerable difference between the two PA level groups. Sedentary time (Mean (median)) was 265 (131) min per day in insufficiently active participants and 211 (118) in sufficiently active participants, respectively.

### 3.1. Levels and Pattern of PA

Of the whole study population, 20.6% reported being insufficiently active (Table 2). The highest fraction of insufficiently active was among patients aged < 55 years. Energy expenditure was negatively correlated to age (*p* < 0.05). Among participants aged 65 years or older most of the energy expenditure was related to recreational activity (53% or 1132 and 68% or 1109 MET min per week in age groups 65–74 and ≥75 years, respectively). Conversely, among T2D participants younger than 55 years, the highest amount of energy expenditure was from work activities (71% or 2558 MET min per week). In all age groups, the lowest amount of energy expenditure was from transport activities.

### 3.2. PA Level in Relation with Glycemic Control

In the study, FPG was significantly lower in the sufficiently active group than in the insufficiently active group (7.10 ± 1.87 mmol/L compared to 8.54 ± 3.64, *p* < 0.001) (Table 3).

Using univariate regression, reaching the WHO recommendation on PA was related with higher odds ratios for controlled plasma glucose OR 2.3 (95%CI 1.4–3.8). On multivariate logistic regression, sufficiently active persons had two-fold higher odds of having controlled plasma glucose (adjusted for age, gender, family history of T2D, WHR, sedentary time, duration of diabetic diagnosis).

## 4. Discussion

The main finding of the present study was that about one fifth of T2D participants did not reach the WHO recommendation on PA for health and the lowest amount of energy expenditure was from travel to and from places. Being sufficiently active was associated with better glycemic management in outpatient clinics.

Our study contributes to the existing knowledge of PA levels assessed by using GPAQ in Vietnam among the general population and another study on diabetic participants in a South-East Asia country [25,26]. The fraction of participants reported being sufficiently active was similar as in a previous cross-sectional study among diabetic patients in lower-middle income countries in Asia [26,27]. GPAQ measures three characteristics of all domains of PA which were the basis of the recommended dose for T2D participants. The GPAQ shows cards and local examples of type and intensity of daily activities suitable for and validated in the Vietnamese context [21]. Additionally, GPAQ has been envisioned as a feasible and cost-effective measurement which has shown a moderate correlation with objective accelerometer assessed moderate to vigorous PA [28]. These results reflected the circumstance that PA in three domains (work, transport and recreational activity) was as common among diabetic participants as in the general population in Vietnam [25].

The findings from this study showed that sufficiently active patients with T2D have a two-fold higher chance of having controlled plasma glucose. Among T2D patients, there was high-certainty evidence that structured exercise training is associated with better HbA1c control [16,29]. Previous review highlighted improvements in HbA1c and reductions in low-density lipoprotein (LDL) as important and well-established finding in PA intervention for diabetic patients [30]. An interesting notion is that interventions with more vigorous aerobic exercise programs resulted in greater reductions in HbA1c. There are some mechanisms that can explain the relationship between PA and glucose control. Insulin sensitivity, which is one of mechanism of diabetes, is improved by moderate to high level of PA [14]. Additionally, the health benefits of aerobic PA include increasing mitochondrial density and cardiac output [31].

One of the key issues of lifestyle change to manage metabolic syndrome is reaching the WHO recommendation on PA for health [6,14]. In line with that assertion, our results showed that self-reported PA level at or above the WHO recommendation for health was related with better WHR management. Our results strengthen the previous notion that sufficient PA can decrease some risk factors for cardiovascular diseases [8,32].

It is now well established that PA can be an effective component in the management of T2D [33,34]. However, the awareness in the general population on the role of PA in prevention and treatment T2D is limited [35]. Encouraging the population to be sufficiently active in all domains of daily living (activity at work, transport to and from places like from home to work and in leisure time) and implementing the PA on prescription are of importance for Vietnam. As part of this mission, an International Physical Activity Prescription Organization (ipapo.org) was started by experts in the PA area from Sweden and Vietnam with the intention to make PA reach everyone [36]. The Vietnamese version book about the PA on prescription, free to download, is provided the basis knowledge for all physicians in daily medical practice. Additionally, participation in PA can be affected and improved by physician’s motivation and by social and environmental support [37,38]. Motorbikes compromised 80-85% of Hanoi’s total road traffic as reported in 2012 as the result of a project supported by the Asian Development Bank [39]. In recent years, the number of cars has increased enormously in Hanoi, while the number of bicycles dropped steeply although rather many sport bicycles can be observed in the traffic of Hanoi nowadays [39]. Therefore, identifying and improving selected environments to produce positive changes in PA (including regularly walking or cycling) are important [40].

A limitation of this study can be that it was conducted at outpatient clinics where many participants had relatively good health and were mobile. Most of the participants who accepted to take part in the study were already active and it might be that more of those who did not come to the clinic were inactive (Hawthorne effect) [41]. It might have led to an overestimation of the prevalence of sufficiently active in the population. This study was conducted in the two hospitals in a big city, which may not be representative for all diabetic patients in Vietnam. The outcomes should be cautiously interpreted in clinical practice. Since the study employs a cross-sectional design, the results showing differences or association may or may not be causally related to sociodemographic and clinical factors and PA level.

## 5. Conclusions

About one fifth of T2D participants did not reach the WHO recommendation on PA for health and the lowest amount of energy expenditure was from transport activities. Being sufficiently active was associated with better glycemic management in outpatient clinics. The findings warrant action plans to increase physical activity in general and in specific active transport, like walking or bicycling, for insufficiently active participants with T2D in Vietnam.

## Figures and Tables

**Table 1:** Characteristics of the study participants.

	Sufficiently Active *N* = 323 (79.4%)	Insufficiently Active *N* = 84 (20.6%)	*p* *
Age (years) ^a^	62.0 ± 9.5	59.4 ± 10.4	0.062
Gender ^b^			
Male	38 (45.2)	184 (57.0)	0.026
Female	46 (54.8)	139 (43.0)	
Duration of T2D diagnosis (years) ^a^	4.5 ± 4.9	2.3 ± 3.5	<0.001
Systolic blood pressure (mmHg) ^a^	121.2 ± 14.7	124.6 ± 17.0	0.074
Diastolic blood pressure (mmHg) ^a^	76.6 ± 7.9	76.2 ± 9.0	0.723
WHR ^a^	0.92 ± 0.05	0.94 ± 0.05	0.028
BMI (kg/m^2^) ^a^	22.3 ± 2.7	22.6 ± 2.7	0.54
Fasting plasma glucose (mmol/L)	7.10 ± 1.87	8.54 ± 3.64	<0.001
Total MET minutes per week ^c^	3380 (2240)	213 (0)	<0.001
Sedentary time (minutes per day) ^b^	211 ± 118	265 ± 131	<0.001

* Unequal variances t-test, ^a^ Mean ± standard deviation; ^b^ n (%); ^c^ Mean (median). BMI, Body Mass Index; T2D, Type 2 diabetes; MET, Metabolic Equivalent of Task; WHR, Waist Hip Ratio.

**Table 2:** Physical activity levels and domains by age groups.

		Age Groups				Total
		<55 (*n* = 98)	55–64 (*n* = 170)	65–74 (*n* = 112)	≥75 (*n* = 27)	
Physical activity levels ^a^						
Insufficiently active	Low ^1^	28 28.6	31 18.2	20 17.9	5 18.5	84 20.6
Sufficiently active *						
	Moderate ^2^	34 34.7	85 50.0	66 58.9	19 70.4	204 50.1
	High ^3^	26 26.5	46 27.1	24 21.4	3 11.1	99 24.4
	Very high ^4^	10 10.2	8 4.7	2 1.8	0 0.0	20 4.9
Physical activity domains (MET minutes per week) ^b^						
Work activity		2558	1230	567	124	
		480	0	0	0	
Transport activity		448	445	446	394	
		0	120	210	0	
Recreational activity (in leisure time)		622	1089	1132	1109	
		0	840	840	840	
Total PA		3627	2764	2145	1628	
		1810	1680	1680	1680	

^a^ n, %; ^b^ Values were expressed as means and medians; * Sufficiently active (reaching the WHO recommendation on PA: ≥600 MET min per week); ^1^ <600 MET min per week; ^2^ 600–2999 MET min per week; ^3^ 3000–7999 MET min per week; ^4^ ≥8000 MET min per week.

**Table 3:** Logistic regression of sufficiently active on controlled plasma glucose.

	Odds Ratios for Controlled Plasma Glucose	95%CI
Model 1	2.30	1.41–3.75
Model 2	2.17	1.32–3.56
Model 3	2.00	1.16–3.46

Model 1: Unadjusted; Model 2: Adjusted by age and gender; Model 3: Adjusted by age, gender, family history of T2D, WHR, sedentary time, duration of diabetic diagnosis.

## Data Availability

The data presented in this study are available on request from the corresponding author. The data are not publicly available due to restrictions of privacy.

## Supplementary Materials

The following are available online at https://www.mdpi.com/1660-4601/18/3/1182/s1, Table S1: data collection form.

## References

[1] World Health Organization (2011). The Top 10 Causes of Death.

[2] Cho N., Shaw J.E., Karuranga S., Huang Y., da Rocha Fernandes J.D., Ohlrogge A.W., Malanda B. (2018). IDF Diabetes Atlas: Global estimates of diabetes prevalence for 2017 and projections for 2045. Diabetes Res. Clin. Pract..

[3] Truong L.D. (2010). Non-Communicable Disease Prevention and Control in Vietnam. The Role of Physical Activity and Lifestyle in NCDs Prevention and Treatment.

[4] Pham N.M., Eggleston K. (2015). Eggleston, Diabetes prevalence and risk factors among Vietnamese adults: Findings from community-based screening programs. Diabetes Care.

[5] Hallal P.C., Andersen L.B., Bull F.C., Guthold R., Haskell W., Ekelund U., Lancet Physical Activity Series Working Group (2012). Global physical activity levels: Surveillance progress, pitfalls, and prospects. Lancet.

[6] Islam S.M.S., Purnat T.D., Phuong N.T.A., Mwingira U., Schacht K., Fröschl G. (2014). Non-Communicable Diseases (NCDs) in developing countries: A symposium report. Glob. Health.

[7] World Health Organization (2014). Global Status Report on Noncommunicable Diseases 2014.

[8] Kyu H.H., Bachman V.F., Alexander L.T., Mumford J.E., Afshin A., Estep K., Veerman J.L., Delwiche K., Iannarone M.L., Moyer M.L. (2016). Physical activity and risk of breast cancer, colon cancer, diabetes, ischemic heart disease, and ischemic stroke events: Systematic review and dose-response meta-analysis for the Global Burden of Disease Study 2013. BMJ.

[9] Laaksonen D.E., Lindström J., Lakka T.A., Eriksson J.G., Niskanen L., Wikström K., Aunola S., Keinänen-Kiukaanniemi S., Laakso M., Valle T.T. (2005). Physical activity in the prevention of type 2 diabetes: The Finnish diabetes prevention study. Diabetes.

[10] Wang Q., Zhang X., Fang L., Guan Q., Gao L., Li Q. (2018). Physical Activity Patterns and Risk of Type 2 Diabetes and Metabolic Syndrome in Middle-Aged and Elderly Northern Chinese Adults. J. Diabetes Res..

[11] Professional Asociations for Physical Activity (2010). Physical Activity in Prevention and Treatment of Disease.

[12] Ekelund U., Tarp J., Steene-Johannessen J., Hansen B.H., Jefferis B., Fagerland M.W., Whincup P., Diaz K.M., Hooker S.P., Chernofsky A. (2019). Dose-response associations between accelerometry measured physical activity and sedentary time and all cause mortality: Systematic review and harmonised meta-analysis. BMJ.

[13] Sluik D., Buijsse B., Muckelbauer R., Kaaks R., Teucher B., Tj A., Overvad K., Amiano P., Ardanaz E., Bendinelli B. (2012). Physical activity and mortality in individuals with diabetes mellitus: A prospective study and meta-analysis. Arch. Intern. Med..

[14] Colberg S.R., Sigal R.J., Yardley J.E., Riddell M.C., Dunstan D.W., Dempsey P.C., Horton E.S., Castorino K., Tate D.F. (2016). Physical activity/exercise and diabetes: A position statement of the American Diabetes Association. Diabetes Care.

[15] World Health Organization (2010). Global Recommendations on Physical Activity for Health.

[16] Bull F.C., Al-Ansari S.S., Biddle S., Borodulin K., Buman M.P., Cardon G., Carty C., Chaput J.P., Chastin S., Chou R. (2020). World Health Organization 2020 guidelines on physical activity and sedentary behaviour. Br. J. Sports Med..

[17] Definition W. (2006). Diagnosis of Diabetes Mellitus and Intermediate Hyperglycemia: Report of a WHO/IDF Consultation.

[18] Van Do V., Jancey J., Pham N.M., Nguyen C.T., Van Hoang M., Lee A.H. (2019). Objectively Measured Physical Activity of Vietnamese Adults With Type 2 Diabetes: Opportunities to Intervene. J. Prev. Med. Public Health.

[19] Armstrong T., Bull F. (2006). Development of the world health organization global physical activity questionnaire (GPAQ). J. Public Health.

[20] Trinh O.T., Nguyen N.D., Dibley M.J., Phongsavan P., Bauman A.E. (2008). The prevalence and correlates of physical inactivity among adults in Ho Chi Minh City. BMC Public Health.

[21] Trinh O.T., Do Nguyen N., Van Der Ploeg H.P., Dibley M.J., Bauman A. (2009). Test-retest repeatability and relative validity of the Global Physical Activity Questionnaire in a developing country context. J. Phys. Act. Health.

[22] World Health Organization (2006). Global Physical Activity Questionaire (Version 2.0): Analysis Guide.

[23] Ainsworth B.E., Haskell W.L., Whitt M.C., Irwin M.L., Swartz A.M., Strath S.J., OBrien W.L., Bassett D.R., Schmitz K.H., Emplaincourt P.O. (2000). Compendium of physical activities: An update of activity codes and MET intensities. Med. Sci. Sports Exerc..

[24] American Diabetes Association (2017). Standard of medical care in diabetes. Diabetes Care.

[25] Van Bui T., Blizzard C.L., Luong K.N., Le Van Truong N., Tran B.Q., Otahal P., Srikanth V., Nelson M.R., Au T.B., Ha S.T. (2015). Physical activity in Vietnam: Estimates and measurement issues. PLoS ONE.

[26] Palermo M., Sandoval M.A. (2016). Assessment of Physical Activity Level among Patients with Type 2 Diabetes Mellitus at the UP–Philippine General Hospital Diabetes Clinic. J. ASEAN Fed. Endocr. Soc..

[27] Ranasinghe D.C., Ranasinghe P., Jayawardena R., Matthews D.R., Katulanda P. (2014). Evaluation of physical activity among adults with diabetes mellitus from Sri Lanka. Int. Arch. Med..

[28] Cleland C.L., Hunter R.F., Kee F., Cupples M.E., Sallis J.F., Tully M.A. (2014). Validity of the global physical activity questionnaire (GPAQ) in assessing levels and change in moderate-vigorous physical activity and sedentary behaviour. BMC Public Health.

[29] Umpierre D., Ribeiro P.A., Kramer C.K., Leitao C.B., Zucatti A.T., Azevedo M.J., Gross J.L., Ribeiro J.P., Schaan B.D. (2011). Physical activity advice only or structured exercise training and association with HbA1c levels in type 2 diabetes: A systematic review and meta-analysis. JAMA.

[30] Zanuso S., Jimenez A., Pugliese G., Corigliano G., Balducci S. (2010). Exercise for the management of type 2 diabetes: A review of the evidence. Acta Diabetol..

[31] Garber C.E., Blissmer B., Deschenes M.R., Franklin B.A., Lamonte M.J., Lee I.M., Nieman D.C., Swain D.P. (2011). American College of Sports Medicine position stand. Quantity and quality of exercise for developing and maintaining cardiorespiratory, musculoskeletal, and neuromotor fitness in apparently healthy adults: Guidance for prescribing exercise. Med. Sci. Sports Exerc..

[32] Warburton D.E., Nicol C.W., Bredin S.S. (2006). Health benefits of physical activity: The evidence. Can. Med. Assoc. J..

[33] Boulé N.G., Kenny G.P., Haddad E., Wells G.A., Sigal R.J. (2003). Meta-analysis of the effect of structured exercise training on cardiorespiratory fitness in Type 2 diabetes mellitus. Diabetologia.

[34] Nguyen C.T. (2017). Independent and joint associations of total physical activity and sedentary time on the risk of type 2 diabetes in adults. Arena J. Phys. Act..

[35] Binh T., Phuong P.T., Nhung B. (2015). Knowledge and associated factors towards type 2 diabetes among a rural population in the Red River Delta region, Vietnam. Rural Remote Health.

[36] Beckvid-Henriksson G., Nguyen T.H., Kilhed J., Nordström A., Svensson S., Tran T.T.H., Van Der Ploeg I., Sundberg C.J. (2018). Implementation and assessment of diverse strategies for physical activity promotion in Vietnam—A case report. J. Sport Health Sci..

[37] Duclos M., Dejager S., Postel-Vinay N., di Nicola S., Quéré S., Fiquet B. (2015). Physical activity in patients with type 2 diabetes and hypertension–insights into motivations and barriers from the MOBILE study. Vasc. Health Risk Manag..

[38] Donahue K.A.T.R.I.N.A., Sloane P.D., Callahan L., Mielenz T. Identifying supports and barriers to physical activity in patients at risk for diabetes. Proceedings of the Annual Meeting of the American Diabetes Association.

[39] Labbé D. (2021). Urban Transition in Hanoi: Huge Challenges Ahead.

[40] Duncan M.J., Spence J.C., Mummery W.K. (2005). Perceived environment and physical activity: A meta-analysis of selected environmental characteristics. Int. J. Behav. Nutr. Phys. Act..

[41] Sedgwick P., Greenwood N. (2015). Understanding the Hawthorne effect. BMJ.

