# Supplementary material for: Physical Activity and Plasma Glucose Control among Diabetic Patients Attending Outpatients Clinics in Hanoi, Vietnam
Source: Int J Environ Res Public Health. 2021 Jan 29;18(3):1182. doi: 10.3390/ijerph18031182 (PMC7908120; doi:10.3390/ijerph18031182)
Supplement: Supplementary file 1 [file ijerph-18-01182-s001.pdf]

DATA COLLECTION FORM

TIME AND LOCATION		ANSWER	
I1	Location		
I2	Interviewer		
I3	Time	└──┴──┘ └──┴──┘ └──┴──┴──┴──┘<br>Date month year	
I4	Sign inform consent	Yes	1
		No	2 **(If No, finish)**
I5	Full name		
I6	Telephone number	└──┴──┴──┘ └──┴──┘ └──┴──┘ └──┴──┴──┴──┘	

STAGE 1: GENERAL INFORMATION				
**C1**	Gender	Male<br>Female	1<br>2	□
**C2**	How old are you?		Year	□□

**D1**	Duration of diabetic diagnosis (year)	
**D2**	Hieght (m)	□,□□
**D3**	Weight (kg)	□□,□
**D4**	BMI (kg/m²)	□□,□
**D5**	Waist circumference (cm)	□□,□
**D6**	Hip circumference (cm)	□□,□
**D7**	WHR	□□,□
**D8**	Systolic blood pressure (mmHg)	□□□
**D9**	Diatolic blood pressure (mmHg)	□□□
**D10**	Fasting plasma glucose (mmol/l)	□□,□

STAGE 2: PHYSCICAL ACTIVITY			
Next I am going to ask you about the time you spend doing different types of physical activity in a typical week. Please answer these questions even if you do not consider yourself to be a physically active person. Think first about the time you spend doing work. Think of work as the things that you have to do such as paid or unpaid work, study/training, household chores, harvesting food/crops, fishing or hunting for food, seeking employment. *[Insert other examples if needed].* In answering the following questions 'vigorous- intensity activities' are activities that require hard physical effort and cause large increases in breathing or heart rate, 'moderate-intensity activities' are activities that require moderate physical effort and cause small increases in breathing or heart rate.			
**Questions**		**Response**	**Code**
**Activity at work**			
1	Does your work involve vigorous-intensity activity that causes large increases in breathing or heart rate like *[carrying or lifting heavy loads, digging or construction work*] for at least 10 minutes continuously? *[INSERT EXAMPLES] (USE SHOWCARD)*	Yes 1	
		No 2 *If No, go to P 4*	P1
2	In a typical week, on how many days do you do vigorous-intensity activities as part of your work?	Number of days └─┘	P2
3	How much time do you spend doing vigorous-intensity activities at work on a typical day?	Hours : minutes └─┴─┘ : └─┴─┘<br>hrs mins	P3 (a-b)
4	Does your work		

	involve moderate-intensity activity that causes small increases in breathing or heart rate such as brisk walking *[or carrying light loads*] for at least 10 minutes continuously? *[INSERT EXAMPLES] (USE SHOWCARD)*	Yes<br>No	1<br>2 *If No, go to P 7*	P4
5	In a typical week, on how many days do you do moderate-intensity activities as part of your work?	Number of days	└─┘	P5
6	How much time do you spend doing moderate-intensity activities at work on a typical day?	Hours : minutes<br>hrs	└─┴─┘ : └─┴─┘<br>mins	P6 (a-b)
**Travel to and from places**				
The next questions exclude the physical activities at work that you have already mentioned. Now I would like to ask you about the usual way you travel to and from places. For example to work, for shopping, to market, to place of worship. [insert other examples if needed]				
7	Do you walk or use a bicycle (*pedal cycle*) for at least 10 minutes continuously to get to and from places?	Yes<br>No	1<br>2 *If No, go to P 10*	P7
8	In a typical week, on how many days do you walk or bicycle for at least 10 minutes continuously to get to and from places?	Number of days	└─┘	P8
9	How much time do you spend walking	Hours : minutes<br>hrs	└─┴─┘ : └─┴─┘<br>mins	P9 (a-b)

	or bicycling for travel on a typical day?		
**Recreational activities**			
The next questions exclude the work and transport activities that you have already mentioned. Now I would like to ask you about sports, fitness and recreational activities (leisure), [insert relevant terms].			
10	Do you do any vigorous-intensity sports, fitness or recreational (*leisure*) activities that cause large increases in breathing or heart rate like [*running or football,]* for at least 10 minutes continuously?<br>*[INSERT EXAMPLES] (USE SHOWCARD)*	Yes 1<br>No 2 *If No, go to P 13*	P10
11	In a typical week, on how many days do you do vigorous-intensity sports, fitness or recreational (*leisure*) activities?	Number of days └─┘	P11
12	How much time do you spend doing vigorous-intensity sports, fitness or recreational activities on a typical day?	Hours : minutes └─┴─┘ : └─┴─┘<br>hrs mins	P12 (a-b)
13	Do you do any moderate-intensity sports, fitness or recreational (leisure) activities that causes a small increase in breathing or heart rate such as brisk	Yes 1<br>No 2 *If No, go to P16*	P13

	walking,(cycling, swimming, volleyball)for at least 10 minutes continuously? [INSERT EXAMPLES] (USE SHOWCARD)		
14	In a typical week, on how many days do you do moderate-intensity sports, fitness or recreational (leisure) activities?	Number of days └──┘	P14
15	How much time do you spend doing moderate-intensity sports, fitness or recreational (leisure) activities on a typical day?	Hours : minutes └─┴─┘ : └─┴─┘ hrs mins	P15 (a-b)
**Sedentary behaviour**			
The following question is about sitting or reclining at work, at home, getting to and from places, or with friends including time spent [sitting at a desk, sitting with friends, travelling in car, bus, train, reading, playing cards or watching television], but do not include time spent sleeping. [INSERT EXAMPLES] (USE SHOWCARD)			
16	How much time do you usually spend sitting or reclining on a typical day?	Hours : minutes └─┴─┘ : └─┴─┘ hrs min s	P16 (a-b)
